# Endoscopic treatment of walled-off pancreatic necrosis complicated with pancreaticocolonic fistula

**DOI:** 10.1007/s00464-018-6032-4

**Published:** 2018-01-17

**Authors:** Mateusz Jagielski, Marian Smoczyński, Krystian Adrych

**Affiliations:** Department of Gastroenterology and Hepatology, Medical University of Gdansk, Smoluchowskiego 17, 80-214 Gdansk, Poland

**Keywords:** Pancreatic fistula, Endoscopic drainage, Transmural drainage, Endoscopic ultrasonography, Walled-off pancreatic necrosis, Acute pancreatitis

## Abstract

**Background:**

Pancreaticocolonic fistulas (PCFs) are serious complication of acute pancreatitis related with high mortality. The aim of this study was to evaluate the efficiency and safety of endoscopic treatment in patients with walled-off pancreatic necrosis (WOPN) complicated with PCF.

**Methods:**

This is a retrospective analysis of results and complications in the group of 226 patients, who underwent endoscopic treatment of symptomatic WOPN between years 2001 and 2016 in the Department of Gastroenterology and Hepatology of Medical University of Gdańsk.

**Results:**

PCF was recognized in 21/226 (9.29%) patients. Transmural drainage was performed in 20/21 (95.24%) patients. Transpapillary drainage was used in 2/21 (9.52) patients. The mean time since the start of endotherapy to the diagnosis of a fistulas was 9 (3–21) days. Fluoroscopic nasocystic tube-check imaging of an existing drain was the initial imaging diagnosis of a PCF in 19/21 (90.48%) patients. The mean duration of endoscopic drainage of WOPN was 39.29 (15–87) days. Procedure-related adverse events occurred in 10/21 (47.62%) patients and most of them were treated conservatively. Three patients required surgical treatment. One patient died during endotherapy. The closure of PCF was confirmed via imaging in 17/21 (80.95%) patients. The average time since the recognition till the closure of PCF was 21 (14–48) days. Complete therapeutic success of WOPN complicated with PCF was reached in 16/21 (76.19%) patients. Long-term success of endoscopic treatment was achieved in 15/21 (71.43%) patients.

**Conclusions:**

Endoscopic treatment of patients with WOPN complicated with PCF is an effective method with an acceptable number of complications. The complete regression of the WOPN may lead to spontaneous closure of pancreaticocolonic fistulas.

**Electronic supplementary material:**

The online version of this article (10.1007/s00464-018-6032-4) contains supplementary material, which is available to authorized users.

Acute pancreatitis is defined as an inflammatory process of the pancreas, surrounding tissues, and distant organs [1, 2]. As a consequence of inflammatory infiltration, fistulization into the neighboring organs may occur [3]. Acute necrotizing pancreatitis can lead to the formation of pancreatic fistulas both in the upper and lower gastrointestinal tract [3, 4]. In different studies, the incidence of gastrointestinal fistulas varies from 4 to 41%, depending on the study population [3–5]. Most pancreatic fistulas occur within the upper gastrointestinal tract, jejunum, and ileum; they can be efficiently treated conservatively [3, 4]. Pancreaticocolonic fistulas (PCFs) are a much more serious complication of acute pancreatitis, and are associated with substantial mortality [4, 6–8]. For many years, surgical intervention was the only recommended method of treatment for patients with PCFs resulting from acute necrotizing pancreatitis [7, 8].

Numerous publications have discussed the surgical treatment of PCFs [7–9]. In the past few years, there have also been a number of publications describing the efficiency of nonsurgical approaches to PCFs caused by acute pancreatitis [3, 4, 10–12]. In the current article, we present the results of endoscopic treatment in 21 patients with walled-off pancreatic necrosis (WOPN) complicated with PCFs. To the best of our knowledge, this is the first publication to present the results of endoscopic treatment of WOPN complicated with PCF that utilized data from a large group of patients.

## Materials and methods

The study was approved by the Ethics Committee of Medical University of Gdansk. All patients gave their informed consent for endoscopic procedures.

### Qualification to study

The indications for endoscopic treatment were determined on the basis of each patient’s clinical picture as well as the results of imaging studies predominantly the abdominal contrast-enhanced computed tomography (CECT). The diagnosis of WOPN was based on the criteria of the 2012 Revision of the Atlanta Classification [1, 2]. Furthermore, the presence of WOPN was confirmed by the appearance of the liquid aspirated from the lumen of necrotic collection (dark brown hue and presence of necrotic debris). Since 2011, endoscopic ultrasonography (EUS) has also been used to confirm the diagnosis of WOPN.

### Exclusion criteria

Patients with WOPN without clinical symptoms related to the presence of necrosis were excluded from this study. We also excluded patients with symptomatic WOPN in whom endoscopic ultrasound showed that the WOPN wall was located more than 15 mm away from the gastrointestinal tract wall, and those in whom endoscopic retrograde pancreatography (ERP) revealed no communication between the main pancreatic duct and the fluid collection.

### Study group

Between 2001 and 2016, a total of 226 patients underwent endoscopic treatment of symptomatic WOPN in the Department of Gastroenterology and Hepatology of the Medical University of Gdansk. Most of the patients had been earlier managed due to acute necrotizing pancreatitis in outside medical centers, but were referred to our department for interventional treatment of WOPN.

### Choice of endoscopic treatment technique

Endoscopic drainage of walled-off pancreatic necrosis has been performed in our center since 2001 [13, 14]. Between 2001 and 2011, 112 patients underwent conventional drainage (CTD) without EUS guidance [13, 14]. After 2011, we used endoscopic ultrasonography to perform transluminal drainage of pancreatic necrosis (EUS-guided drainage) [14]. Between 2011 and 2016, EUS-guided drainage was performed in 114 patients.

Transmural drainage was attempted in all patients with symptomatic WOPN. Drainage was not performed if the distance between the wall of the fluid collection and the gastrointestinal wall exceeded 15 mm. Among the patients who did not undergo transmural drainage, those in whom ERP revealed a leak of contrast medium into the necrotic collection were considered eligible to undergo transpapillary drainage. Furthermore, several patients with incomplete regression of WOPN after a transluminal procedure, and in whom a communication between the main pancreatic duct and the inside of the fluid collection was observed during endoscopic pancreatography, underwent additional transpapillary drainage.

### Description of procedures

The techniques of endoscopic drainage of WOPN used in our medical center were discussed in detail in our previous publications [13, 14].

Between 2001 and 2011, endoscopic procedures were performed with the use of Pentax ED2485K and Pentax ED3440T models of duodenoscope, and in subsequent years (2011–2013)—with Pentax ED3490TK and Pentax EG3870UTK. All endoscopic interventions were performed under deep sedation (pethidine with either diazepam or midazolam). Since 2011, the place of fistulotomy was chosen under EUS guidance. Between 2001 and 2011 (conventional drainage), fistulotomy was performed on the top of the largest protuberance of the necrotic collection into the gastrointestinal wall (65 patients). When no protuberance was visible, the determination of the necrogastrostomy or necroduodenostomy site was made with the help of fluoroscopy after administration of contrast medium either via the duodenal papilla (in the presence of a main pancreatic duct leak) in 32 patients or through a percutaneous drain in 15 patients. Enterostomy was performed with a 7 French fistulotome (Huibregtse Triple Lumen Needle Knife HPC-3, Wilson-Cook) or a Giovannini cystostome (Cystotome CST-10, Wilson-Cook). The opening between the lumen of the gastrointestinal tract and the lumen of the necrotic collection was widened with the use of a “bougie” type dilator (Soehendra Biliary Dilation Catheters SBDC-8.5, SBDC-10, Wilson-Cook) or a high-pressure balloon (8 or 20 mm, Boston Scientific). A 7 French or 8 French nasocystic drain (Balton or Wilson-Cook) and several “double-pigtail” (7 French/8.5 French stents, Wilson Cook/Mar Flow) or 10 French stents by Wilson Cook were inserted into the cavity lumen of the collection.

### Drainage system

The necrotic collection was irrigated with saline solution (60–200 ml) through a nasocystic drain every 2 h during the first 48 h and every 4 h in the subsequent days. Before the procedure all patients received antibiotics (ciprofloxacin or ceftriaxone with metronidazole). Prophylactic antibiotic therapy was continued for 2 weeks. In the presence of clinical symptoms indicating infection of the collection, antibiotic therapy was prolonged or modified in accordance with the results of microbial culture of fluid from the collection. If there was a clinical suspicion of suboptimal drainage, the position of drains was changed or another necrogastrostomy or necroduodenostomy in a new location was performed or a nasocystic drain was introduced through the duodenal papilla into the cavity of the collection via the main pancreatic duct disruption.

### Assessment of therapeutic effect

The size of WOPN was monitored every 7 days by transabdominal ultrasonography. Contrast-enhanced CT was performed to confirm complete regression of the collection. Drains were removed after complete regression of the necrotic collection.

### Endoscopic retrograde pancreatography (ERP)

In cases of a main pancreatic duct (MPD) leak, sphincterotomy was performed (Olympus FlowCut KD-301Q0725 sphincterotome) and a pancreatic stent was inserted into the main pancreatic duct (5–10 French, Geenen, Zimmon Pancreatic Stent, Wilson-Cook or Mar Flow). The transpapillary pancreatic stents were exchanged after 3, 6, and 12 months until no leakage of contrast outside the duct could be demonstrated.

In patients with active transpapillary drainage, after sphincterotomy performed during ERP the main pancreatic duct was mechanically dilated with a “bougie” type dilator (7 French to 10 French, Wilson-Cook). The nasocystic drain and pancreatic stent were placed through the duodenal papilla. The distal tip of nasocystic drain was deployed within the necrotic cavity.

### Definitions

Pancreaticocolonic fistula (PFC) was defined as pathological communications that connect lumen of the colon with the lumen of necrotic collection or main pancreatic duct.

Complete regression of the collection was defined as disappearance of clinical symptoms and a decrease of the collection’s diameter to less than 3 cm.

Closure of a pancreaticocolonic fistula (PCF) was defined as a lack of visualization of previously documented communication between the lumen of the colon and the lumen of necrotic collection or main pancreatic duct on follow-up imaging studies.

Successful endoscopic treatment of pancreatic duct disruption was defined as the absence of contrast medium leakage outside the main pancreatic duct during ERP in patients with established MPD disruptions, in whom a pancreatic stent had been inserted into the MPD.

Complete therapeutic success was defined as the complete regression of the collection, closure of the PCF, and the successful endoscopic treatment of pancreatic duct disruption.

Long-term success was defined as the absence of notable symptoms, complete regression of the collection, no recurrence of MPD disruption, and no recurrence of PCF on follow-up imaging studies performed after a period of time since the completion of active drainage.

### Statistical analysis

All statistical calculations were performed with use of the data analysis software STATISTICA version 10.0 (StatSoft Inc., Tulsa, OK, USA; as licensed for the Medical University of Gdansk). Quantitative variables were characterized by arithmetic means, standard deviation, minimal and maximal values (range), and 95% confidence interval (CI). Qualitative data were presented by means of numbers and percentage.

## Results

Spontaneous PCFs were found in 21 of 226 (9.29%) patients with symptomatic WOPN (Table 1*)*. None of the patients included in the study had undergone any invasive radiologic or surgical intervention before the diagnosis of PCF. In all of our patients, the PCF was discovered during endoscopic treatment. The mean time from the start of endotherapy to the diagnosis of a pancreaticocolonic fistula was 9.23 days [SD 4.36; range 3–21 days]. Additional intra-abdominal fistulas were not found in any patient. A pancreaticopleural fistula was recognized in one of the patients with PCF.

**Table 1:** Characteristics of the patients with walled-off pancreatic necrosis complicated with pancreaticocolonic fistula

	All patients (*n* = 21)
Age, mean, (SD), [range]	51.24 (11.6) [33–81]
Sex, *n* men (%)	17 (80.95%)
Etiology, *n*, (%)	
Alcoholic	14 (66.67%)
Nonalcoholic	7 (33.33%)
WOPN size (cm), mean, (SD), [range]	17.56 (5.1) [8.4–33.0]
WOPN type^a^, *n*, (%)	
Pancreatic parenchymal necrosis alone	19 (90.48%)
Peripancreatic necrosis alone	1/21 (4.76%)
Both pancreatic and peripancreatic necrosis	1/21 (4.76%)
Time from the acute bout of pancreatitis (days), mean, (SD), [range]	104 (52.3) [38–189]
WOPN localization, *n*, (%)	
Pancreatic body and tail	18 (85.71%)
Pancreatic tail	3 (14.29%)
Main indication to start endotherapy, *n*, (%)	
Infected necrosis	15 (71.43%)
Abdominal pain	19 (90.48%)
Gastrointestinal obstruction	13 (61.90%)
Weight loss	7 (33.33%)

^a^The type of necrosis was stated basing on contrast-enhanced computed tomography (CECT)

**Fig. 1:**
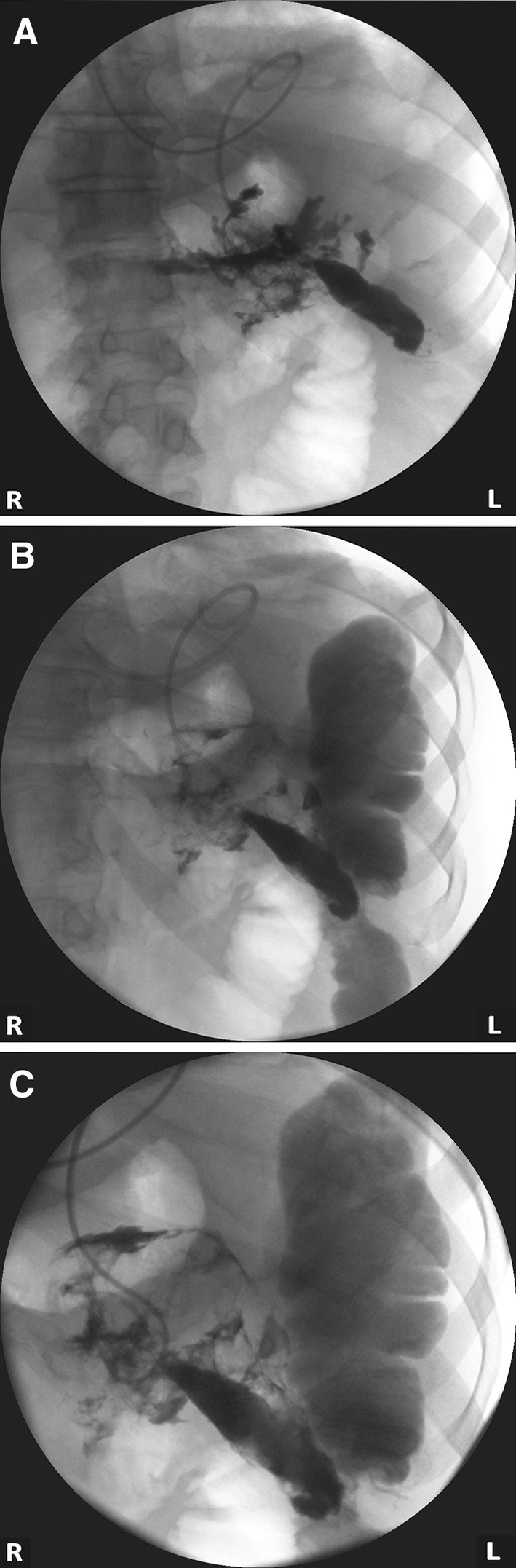
**A**–**C**. Endoscopic transmural (transgastric) drainage of WOPN. The contrast applied through the nasal drain filled the necrotic collection, showing pancreaticocolonic fistula. (*L* left side of patient, *R* right side of patient)

**Fig. 2:**
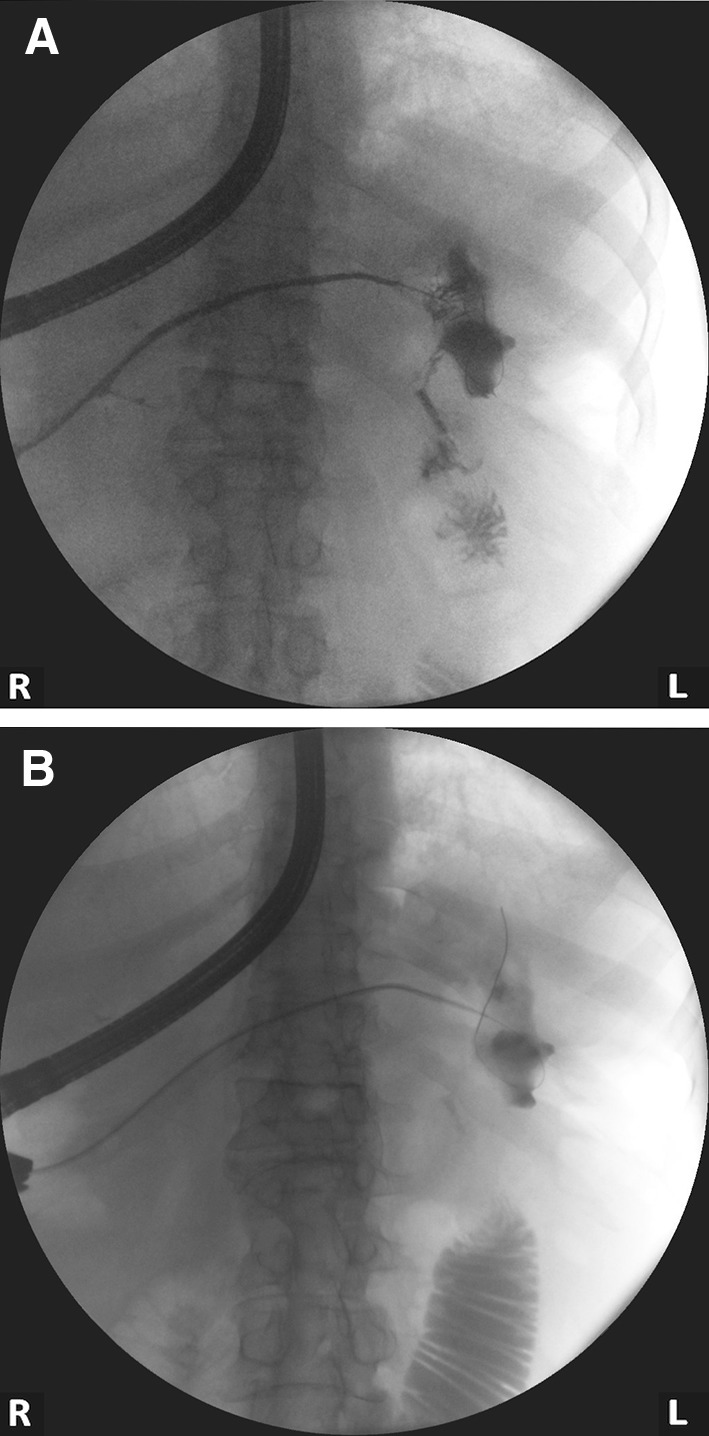
**A, B**. Endoscopic retrograde pancreatography in the patient with WOPN. Applied contrast filled the main pancreatic duct with the visible complete duct disruption in the tail of pancreas. The contrast is leaking to the necrotic collection through the disruption. Pancreaticocolonic fistula with visible leakage to the lumen of colon is also well visible. (*L* left side of patient, *R* right side of patient)

**Fig. 3:**
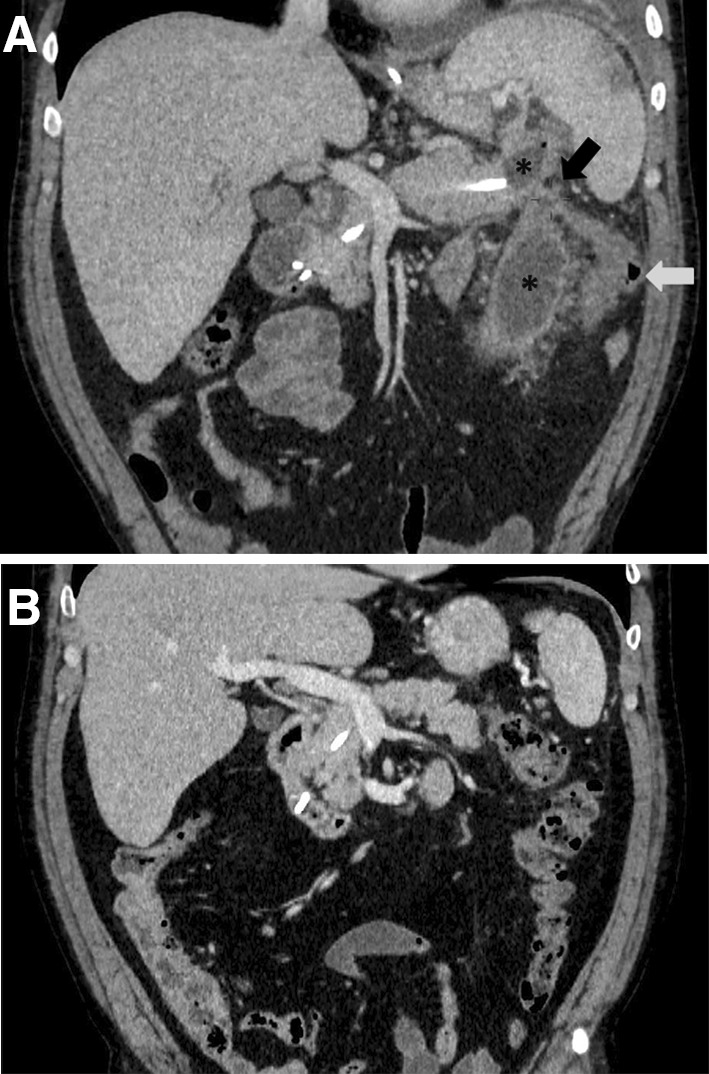
**A** Abdominal contrast-enhanced computed tomography (CECT) done during the endoscopic treatment (video 1) showed a pancreaticocolonic fistula (red arrow) between the walled-off pancreatic necrosis cavity (blue stars) and the colon lumen (green arrow) in the area of splenic flexure. Nasal drain 7 French along with pancreatic endoprosthesis 7 French was inserted to the main pancreatic duct through the major duodenal papilla (active transpapillary drainage). (Color figure online)

Fluoroscopic nasocystic tube-check imaging (Fig. 1A–C) of an existing drain was the initial imaging diagnosis of a PCF in 19 of the 21 patients (90.48%) in whom spontaneous PCFs were recognized. PCF was discovered during the ERP (Fig. 2A, B) in 2 of the 21 patients (9.52%). In all patients, the presence of fistulas was confirmed by CECT (Fig. 3). All PCFs were found in the left colon.

Symptoms likely related to the presence of PCF were reported in 12 of 21 patients (57.14%). Melena was noted in seven patients, while hematochezia with diarrhea was observed in three patients. Sepsis was diagnosed in two patients during the course of endoscopic drainage.

Endoscopic treatment of WOPN was started in all 21 patients, and was completed in 17 of 21 (80.95%) patients (Table 2*)*. Four patients did not complete endotherapy. Two patients underwent surgical drainage of WOPN (one with collection perforation and one with sepsis). One patient required surgical treatment of PCF. One patient died during treatment due to bleeding from splenic artery pseudoaneurysm.

**Table 2:** The results of treatment of 21 patients with WOPN complicated with PCF

	No. patients	%
Total amount of patients	21	100
Complete regression of necrotic collection—efficiency of endoscopic treatment of WOPN	17/21	80.95
Closure of PCF—efficiency of endoscopic treatment of PCF	17/21	80.95
Surgical treatment of WOPN	2/21‡	9.53
Surgical treatment of PCF	1/21	4.76
Mortality	1/21	4.76
Successful endoscopic treatment of pancreatic duct disruption	14/15	93.33
Complete therapeutic success of WOPN complicated with PCF	16/21	76.19
Complications of endotherapy	10/21	47.62
Conservative treatment of complications	9/10	
Surgical treatment of complications	1/10^a^	
The recurrence of WOPN	6/ 21	28.57
Recurrent WOPN treated endoscopically	5/6	
Recurrent WOPN treated surgically	1/6	
Long-term success of endoscopic treatment of WOPN complicated with PCF	15/21	71.43

Detailed description along with explanations of the definitions is in the text of publication

^a^One patient required surgical treatment of endotherapy complications. Surgical drainage of WOPN was done during the procedure

Infection of the WOPN was confirmed by microbial culture in 15 of 21 patients (71.43%). The most common pathogens cultured were Escherichia coli and *Enterococcus faecalis*.

Transmural access was used in 20 of the 21 (95.24%) patients (transgastric in 18 patients, transduodenal in two). Transpapillary drainage (Video 1) was used in two patients. Transpapillary drainage was the only way of gaining access to the necrotic collection in only one patient. Transpapillary drainage was combined with transmural (transgastric) drainage in another patient. No patient in our study underwent percutaneous drainage (PCD).

Procedure-related adverse events occurred in 10 of 21 patients (47.62%). Eight patients required packed red blood cells transfusions, due to gastrointestinal bleeding. One patient died because of bleeding from a splenic artery pseudoaneurysm. Surgical treatment of endotherapy complications was necessary in patient with perforation of the wall of the necrotic collection.

ERP was performed in 18 of 21 patients (85.71%). Main pancreatic ductal leak was seen in 15 patients. Partial disruption of the pancreatic duct was observed in 13 of the 15 patients, while complete disruption was diagnosed in the remaining two patients. A fragment of the main pancreatic duct was contrasted without a leak of contrast medium in 2 patients, and pancreatic duct was found to be normal in just one patient. Transpapillary pancreatic stents were inserted in all patients with pancreatic duct disruptions.

The mean duration of endoscopic drainage of WOPN was 39.29 days [SD 18.23; range: 15–87 days]. The mean number of procedures was 6.14 [SD 4.16; range 4–23]. Complete regression of the collection was seen in 17 of the 21 patients (80.95%).

The closure of PCF was confirmed by imaging in 17 of the 21 patients (80.95%). The average time from the diagnosis to the closure of PCF was 21.12 days [SD 16.22; range 14–48 days].

Successful endoscopic treatment of pancreatic duct disruption was achieved in 14 of the 15 patients (93.33%). The mean duration of the main pancreatic duct stenting was 128 days [SD 112.45; range 69–354 days]. One patient continued to undergo endotherapy for an additional amount of time, due to the complete disruption of the pancreatic duct.

Complete therapeutic success of WOPN complicated with PCF was achieved in 16 of the 21 patients (76.19%).

The mean follow-up period was 30 months [range 15–84]. The recurrence of WOPN was observed in six of the 21 (28.57%) patients during follow-up. No PCFs were found in any of the patients with recurrence of pancreatic fluid collection. Five patients from this group underwent repeat endoscopic therapy. One patient with recurrence of collection was treated surgically. Long-term success of endoscopic treatment of WOPN complicated with PCF was achieved in 15 of the 21 patients (71.43%).

## Discussion

Colonic necrosis, fistula, stricture, and hemorrhage are considered to be uncommon, but potentially lethal, consequences of acute necrotizing pancreatitis [9, 15]. PCFs appear in 8% of patients with acute pancreatitis [4]. PCFs are the most common form of gastrointestinal fistula, followed by duodenal fistula [4]. The presence of fistulas in our study was confirmed in 9.29% of patients with WOPN due to acute necrotizing pancreatitis. Additional intra-abdominal fistulas were not observed in any patients in our study.

In the current literature, there are several theories concerning colonic involvement in the course of acute necrotizing pancreatitis [7, 8]. They include direct causes (local erosion of colon by digestive enzymes) as well as indirect causes (colon ischemia due to vascular thrombosis, compression of mesenteric arteries, and disseminated intravascular coagulation) [7, 8]. According to majority of authors, the most important mechanism of colonic pathology in acute pancreatitis is spread of pancreatic enzymes and arising necrosis of surrounding tissues [8]. Described mechanism mainly concern early phase of acute pancreatitis. It seems that PCFs appear in early phase of severe acute pancreatitis, often coexisting with colonic necrosis. These patients are usually in severe clinical condition with sepsis symptoms, which significantly increases the mortality. Then surgical procedure remains the treatment of choice. The average time from the onset of acute pancreatitis until endoscopic intervention in our paper was 104 days, while the average time from the start of endotherapy to the diagnosis of a pancreaticocolonic fistula was nine days. Despite the usual timing of PCF appearance, PCFs in our study were considered to be late complications of acute necrotizing pancreatitis. This may explain the good clinical condition of most of the patients, as well as the positive long-term results achieved with endotherapy. In our study, PCFs were caused by spontaneous fistulization of WOPN into the lumen of the colon in the late phase of acute necrotizing pancreatitis.

PCFs can also be an adverse complication of interventional treatment of acute necrotizing pancreatitis, such as percutaneous drainage PCD of pancreatic fluid collections [16, 17]. None of the patients described in our paper underwent any interventional procedure (including PCD) prior to the beginning of endoscopic treatment and diagnosis of PCF.

All the fistulas identified in our study were located in the left part of the colon, which was related to the location of the necrotic collections within the body and tail of the pancreas. We hold the view that patients with distal necrosis require particular attention with regard to the possible presence of colonic fistulas. The diagnosis of PCF is considered to be difficult due to unspecified symptoms such as diarrhea, fever, hematochezia, and abdominal pain [10, 18–20]. As per our observations, the PCF should be suspected in patients with acute necrotic pancreatitis complicated by gastrointestinal bleeding, often with sudden deterioration of the general condition, or those with septic shock. No symptoms concerned with the presence of fistula may manifest in other parts of the patients.

Another difficulty in the recognition of PCF comes from the lack of sufficiently sensitive and specific imaging techniques. Endoscopic retrograde pancreatography, magnetic resonance cholangiopancreatography, colonography with barium enema, or computed tomography can all be used for the diagnosis [21–25]. The advantage of ERP over other imaging modalities is the added benefit of pancreatic stent placement in order to bridge the disruption site [26, 27]. Importantly, in only two out of 21 patients with a PCF was this complication discovered during the original ERP. Ultimately, fluoroscopic nasocystic tube-check imaging was revealed to be, in our study, the best method of diagnosing of PCFs (in 19 of the 21 patients). All of the fistulas were confirmed by abdominal CECT. This proves that CECT is suitable for confirming the presence of PCFs. No other imaging techniques of capable of visualizing PCFs were used in the patients in our study.

In our study, primarily infected walled-off pancreatic necrosis was diagnosed in 15 of the 21 patients. This may be due to the fact that, during endotherapy, the procedure uncovered signs of spontaneous fistulization and the creation of PCF in some of the patients who presented with initially sterile necrotic collection. PCF in this group of patients might be a consequence of ineffective endoscopic drainage, related to insufficient access to necrotic collection (particularly with transpapillary drainage). This may also partially explain the fact that no PCF was discovered during the first endoscopic procedure: the average time from the start of endotherapy to the diagnosis of fistula was nine days. However, in our opinion, the delayed diagnosis of PCF is primarily caused by with diagnostic difficulties and a lack of characteristic symptoms specific for a PCF.

PCFs are serious complications of acute pancreatitis associated with high morbidity [7–9, 11], due to accompanying septic complications and hemorrhage. Surgery is often the treatment of choice [9], particularly in the case of septic complications or hemorrhage. Globally, endoscopic treatment has been found to be an accepted and common method of WOPN therapy [13, 14], as well as a treatment for main pancreatic duct disruptions that are consequences of acute necrotizing pancreatitis [26, 27]. Endoscopic drainage is also an alternative to other minimally invasive methods of treatment of pancreatic necrosis [13, 14]. Several reports have described the efficiency of various minimally invasive techniques for the treatment of pancreatic fluid collections complicated with PCF [4, 10, 28]. Despite this, in the literature there are a few case reports available on the efficacy of endotherapy as the only method of minimally invasive treatment of patients with WOPN complicated with PCF [28, 29]. To the best of our knowledge, the current study is the first to present results of endoscopic treatment of WOPN complicated with PCF in a large group of patients.

Howell et al. described successful transmural endoscopic drainage of infected pancreatic fluid collections complicated with PCF [29], while Fujii et al. demonstrated the efficacy of transpapillary drainage in the treatment of PCF [28]. However, both of these publications are case reports [28, 29].

So far, there have been only two studies that demonstrated the efficacy of minimally invasive treatment of pancreatic fluid collection complicated with PCF (as a consequence of severe acute pancreatitis) in a large group of patients [4, 10]. PCD was performed in 20 patients with pancreatic fluid collections complicated with PCF in a study by Heeter et al. [10]. Endoscopic transmural drainage combined with PCD (dual-modality drainage [DMD]) was applied in the same study in three patients [10]. The success of a nonsurgical method of treatment of pancreatic fluid collections complicated by PCFs was noted in the study by Heeter et al. in 15 of the 20 (75%) patients [10]. Another study of patients with PCFs and with infected pancreatic or peripancreatic necrosis treated by PCD or continuous negative pressure irrigation was published by Jiang et al. [4]. In this study, endoscopic drainage was not done in any of the patients [4]. Conversely, in the study by Jiang et al., 21 of the 72 (29.2%) colonic fistulas were successfully treated with the use of PCD [4].

In our study, successful treatment was achieved in 17 of 21 (80.95%) patients with WOPN complicated with PCF. The recurrence of WOPN was observed in 6 of 21 (28.57%) patients during a follow-up. Five patients from this group underwent endoscopic therapy again. Long-term success was achieved in 15 of 21 (71.43%) patients. Comparing the results of treatment presented in this publication with other publications produced in our medical center, in which we did introduce the results of endotherapy of patients with WOPN without PCF [13, 14], we can easily conclude that the endoscopic treatment of patients with WOPN complicated with PCF is related with worse efficiency, higher rates of complication, and a larger amount of recurrent collections. It seems that the presence of a higher amount of recurrent collections in patients with PCF is related with fistula maintenance, even despite the fact that we were unable to visualize it during the imaging examinations. This is also likely due to the lack of efficient and precise imaging techniques for PCFs.

In our study, PCFs provided additional access to the area of WOPN that enabled the flow of necrotic content into the colon during active drainage. Baron et al. described the use of endoscopic necrosectomy performed through the fistula between the necrotic collection and the colon [30]. Endoscopic necrosectomy should be performed particularly in case of inefficiency of active endoscopic drainage in patients with pancreatic necrosis [31, 32]. Perhaps endoscopic necrosectomy would increase efficiency and safety of treatment of described patients WOPN complicated with PCF. In our medical center, endoscopic necrosectomy under fluoroscopic guidance was performed when the following criteria was met—lack of clinical effect or infection of necrotic collection despite the active drainage and large amount of necrotic tissues in fluoroscopic and endosonographic image [33].

There are reports in the literature that the conservative treatment of PCFs can lead to their spontaneous closure, but that is reserved for stabile patients only [9, 11]. The strategy of conservative treatment was not applied in any of the patients in our study. The reason for this was that no PCF was identified prior to the start of endotherapy. All of the fistulas in our study were discovered only during endoscopic treatment of WOPN.

The main limitations of our study are its nonrandomized, retrospective character and its use of a highly selected group of patients from a single center. Conversely, the fact all the endoscopic procedures were completed by one endoscopist allows for a reliable comparison of results of the endotherapy. Another limitation of the current study is that we did not use self-expandable metal stents (SEMS), which decrease the number of procedures and duration of WOPN treatment, which increases the effectiveness of therapy [32, 34]. We hold the view that further studies (specifically prospective randomized, controlled trials) concerning the efficacy of endotherapy of WOPN complicated with PCF are necessary. However, we find the randomized, prospective study of this group of patients to be very difficult.

Our study results prove that WOPN complicated with PCF may be effectively treated with the use of minimally invasive techniques. The complete regression of WOPN may lead to spontaneous closure of PCFs. The choice of the method of access to the pancreatic necrosis, as well as the selection of minimally invasive technique, should depend on the extent of necrosis as well as the experience and capabilities of the medical center.

In conclusion, the present study shows that endotherapy is an effective method of treatment in patients with WOPN complicated with PCF and can be used as a sole treatment modality. Notably, it demonstrated an acceptable rate of complications. In case of endotherapy failure, the surgical treatment remains the treatment of choice.

## Electronic supplementary material

Below is the link to the electronic supplementary material.

Video 1. Drainage system based on multiple access to necrotic collection. Contrast injected via the nasal drain filled walled-off pancreatic necrosis with a leak through the pancreaticocolonic fistula in the region of splenic flexure into the colon lumen. Video 1 is inverted compared to the traditional ERCP view, since the patient is laying on the back during the record. (WMV 2166 KB)
